# Physiological Function of Gastrin-Releasing Peptide and Neuromedin B Receptors in Regulating Itch Scratching Behavior in the Spinal Cord of Mice

**DOI:** 10.1371/journal.pone.0067422

**Published:** 2013-06-24

**Authors:** Devki D. Sukhtankar, Mei-Chuan Ko

**Affiliations:** 1 Department of Pharmacology, University of Michigan Medical School, Ann Arbor, Michigan, United States of America; 2 Department of Physiology and Pharmacology, Wake Forest University School of Medicine, Winston-Salem, North Carolina, United States of America; University of Missouri, United States of America

## Abstract

Pruritus (itch) is a severe side effect associated with the use of drugs as well as hepatic and hematological disorders. Previous studies in rodents suggest that bombesin receptor subtypes i.e. receptors for gastrin-releasing peptide (GRPr) and neuromedin B (NMBr) differentially regulate itch scratching. However, to what degree spinal GRPr and NMBr regulate scratching evoked by intrathecally administered bombesin-related peptides is not known. The first aim of this study was to pharmacologically compare the dose-response curves for scratching induced by intrathecally administered bombesin-related peptides versus morphine, which is known to elicit itch in humans. The second aim was to determine if spinal GRPr and NMBr selectively or generally mediate scratching behavior. Mice received intrathecal injection of bombesin (0.01–0.3 nmol), GRP (0.01–0.3nmol), NMB (0.1–1nmol) or morphine (0.3–3 nmol) and were observed for one hour for scratching activity. Bombesin elicited most profound scratching over one hour followed by GRP and NMB, whereas morphine failed to evoke scratching response indicating the insensitivity of mouse models to intrathecal opioid-induced itch. Intrathecal pretreatment with GRPr antagonist RC-3095 (0.03–0.1 nmol) produced a parallel rightward shift in the dose response curve of GRP-induced scratching but not NMB-induced scratching. Similarly, PD168368 (1–3 nmol) only attenuated NMB but not GRP-induced scratching. Individual or co-administration of RC-3095 and PD168368 failed to alter bombesin-evoked scratching. A higher dose of RC-3095 (0.3 nmol) generally suppressed scratching induced by all three peptides but also compromised motor function in the rotarod test. Together, these data indicate that spinal GRPr and NMBr independently drive itch neurotransmission in mice and may not mediate bombesin-induced scratching. GRPr antagonists at functionally receptor-selective doses only block spinal GRP-elicited scratching but the suppression of scratching at higher doses is confounded by motor impairment.

## Introduction

Itch (pruritus) is an unpleasant sensation, which provokes the desire to scratch. Itch is a dominant symptom of several medical conditions such as cholestasis, atopic dermatitis and uremia [1], [2]. Chronic itch, which typically lasts more than six weeks, has a substantial impact on the quality of life [3]–[5]. Despite being a significant medical burden, the effective management of pruritus poses a major challenge due to the lack of broad-spectrum antipruritic drugs. Also, commonly prescribed antipruritic drugs such as topical emollients and antihistamines fail to relieve chronic itch [2], [6]. Such hurdles are largely due to the poor understanding of the biological mechanisms that drive the sensation of itch. Therefore, more preclinical research is warranted in order to identify the receptors that mediate itch and to characterize potential antipruritic drugs.

Studies in animal models using different types of pruritogens have improved the knowledge of biological modulators of itch. One such pruritogen is bombesin, which when centrally administered, elicits profound scratching across diverse animal species [7]–[10]. Bombesin is a tetradecapeptide originally isolated from frog skin [11] and causes scratching activity in rodents that is much more intense than other pruritogens such as gastrin-releasing peptide (GRP), neuromedin B (NMB), substance P and morphine [9], [10], [12]–[14]. Bombesin has a relatively high affinity for the bombesin receptor subtypes: gastrin-releasing peptide receptor (GRPr) and neuromedin-B receptor (NMBr) [15]. Previous studies using GRPr mutant mice or the GRPr antagonist have shown attenuated scratching in response to intradermally injected pruritogens such as chloroquine and protease activated receptor 2 [16]. Interestingly, the GRPr antagonist also blocked intrathecal morphine evoked scratching in mice [17]. Thus, GRPr is one of the important mediators of itch and GRPr antagonists may have the potential to be effective antipruritics. This notion can be further strengthened by demonstrating the role of GRPr in regulating scratching evoked by spinally administered pruritogens.

Recent work from our lab revealed a pharmacological basis for the supraspinal actions of bombesin, GRP and NMB to induce scratching in rats [18]. We demonstrated that at the supraspinal level, GRPr and NMBr independently mediate scratching. In addition, bombesin-induced scratching is not mediated by GRPr and NMBr but an unidentified subset of receptors. To what degree GRPr and NMBr in the spinal cord regulate scratching evoked by intrathecally administered bombesin-related peptides is not known. Understanding the selectivity and interaction between bombesin-related peptides and their receptors is crucial for the development of GRPr and NMBr antagonists as potential antipruritic drugs.

Itch is also the most common side effect of spinally administered mu-opioid receptor (MOP) agonists like morphine. This type of itch can be severe and hampers the quality of analgesia [19]–[21]. Although intrathecal morphine induced scratching is previously reported in rodents, whether or not morphine can elicit profound or measurable scratching in rodents that can be distinguished from intrathecal injection of its vehicle is somewhat controversial [9], [22]. The magnitude and duration of scratching induced by intrathecal morphine at the antinociceptive doses is not well characterized in mice. In particular, it is not known how intrathecal morphine induces scratching compared to the bombesin-related peptides in mice. Such pharmacological comparisons are important to gain insights into the receptor mechanisms such as the possible interactions between mu-opioid and bombesin-family receptors to regulate scratching behaviors, knowing which will further facilitate the cause-specific treatment of chronic itch.

Therefore, the main goals of this study were (a) to pharmacologically characterize the dose-response curves and duration of scratching induced by intrathecally administered bombesin-related peptides such as bombesin, GRP and NMB as well as morphine in mice and, (b) to determine whether GRPr and NMBr in the spinal cord independently or mutually regulate scratching evoked by bombesin-related peptides using the selective GRPr and NMBr antagonists.

## Methods

### Animals

Male NIH-Swiss mice weighing 25–30 g were used (Harlan, IN). Mice were housed five per cage with free access to food and water and 12∶12 h day-night cycle under the standard laboratory conditions.

Ethics statement: This study was carried out in strict accordance with the recommendations in the Guide for the Care and Use of Laboratory Animals of the National Institute of Health (Bethesda, MD). The protocol was approved by the University Committee on the Use and Care of Animals at the University of Michigan (Ann Arbor, MI) (protocol number: PRO00004606). All efforts were made to minimize the suffering.

### Drug Administration

Bombesin, GRP, NMB (R&D Systems, MN), RC-3095 (Sigma-Aldrich, MO) and morphine (National Institute on Drug Abuse, MD), were dissolved in sterile water. PD168368 (R&D Systems, MN) was dissolved in 1∶1:8 ratio of dimethyl sulfoxide, Tween 80 and sterile water. All drugs were administered intrathecally in the volume of 5 μl as previously described [23]. Briefly, the mouse was secured by a firm grip on the pelvic girdle. Drugs were injected by lumbar puncture between L5/L6 vertebrae using the 30-guage needle attached to a 10 μl Hamilton syringe. Mice in the control group received intrathecal injection of the vehicle.

### Behavioral Analyses

#### Scratching

Mice were habituated for 20 min in plastic cages with small amount of bedding. Scratching behavior was quantified as the number of scratching bouts. One scratching bout was defined as lifting of the hind limb, directing it toward the flank area to scratch and then placing it back on the floor, irrespective of the number of strokes that took place during that movement. All mice were observed for 1 h and number of scratching bouts at 10 min intervals was counted.

#### Rotarod

Mice were tested on the rotarod (IITC, CA) for the assessment of their motor function. The rotarod consisted of five textured drums of 1.25 cm diameter. Total time that the mouse was able to remain on the rotating drum was recorded. Training consisted of habituation during which the mice were acclimatized to the rotarod at 5 rpm for 180 seconds and training during which they were allowed to remain on the rotarod at 10 and 15 rpm for 180 sec. On the test day, all mice were tested at 15, 20, 25 and 30 rpm for 180 sec and 10 min rest period was allowed between each trial.

### Experimental Design

All mice were randomly assigned to each dosing condition (n = 6 per group) and observed by experimenters blinded to these conditions. The first part of the study was conducted to determine the magnitude and duration of scratching induced by bombesin-related peptides and morphine. Bombesin (0.01–0.3 nmol), GRP (0.01–0.3 nmol), NMB (0.1–1 nmol) or morphine (0.3–3 nmol) were intrathecally administered. Immediately after the drug administration, the number of scratching bouts was measured in 10 min intervals for 1 h. In the second part of the study, effects of GRPr and NMBr antagonists on GRP, NMB and bombesin-induced scratching were determined. All antagonists were administered intrathecally as a 10 min pretreatment. Shift in the dose response curve for GRP-induced scratching was determined following administration of the selective GRPr antagonist RC-3095 (0.03–0.3 nmol). Shift in the dose response curve for NMB-induced scratching was determined following administration of the selective NMBr antagonist PD168368 (1–3 nmol). Scratching bouts were measured as previously described. The doses of antagonists which caused the maximum (10-fold) parallel rightward shift in the dose response curve for GRP or NMB were chosen for further studies. RC-3095 (0.1 nmol) was administered as a pretreatment to NMB or bombesin whereas PD168368 (3 nmol) was administered as a pretreatment to GRP or bombesin. In addition, a separate group of mice injected with bombesin were pretreated with a single solution containing 0.1 nmol of RC-3095 and 3 nmol of PD168368. Dose response curve for the effect of RC-3095 on GRP-induced scratching showed that 0.3 nmol of RC-3095 did not cause a parallel right ward shift but instead a general suppression of scratching induced by GRP, NMB, and bombesin. Hence, in order to determine whether this effect was due to the inhibition of motor behavior, in the third part of the study, mice were tested on the rotarod 10 min after the intrathecal injection of 0.3 nmol RC-3095.

### Data Analysis

All data are represented as mean values (mean ± SEM) calculated from individual animals for all behavioral endpoints. Data for the time course representing the number of scratching bouts at 10 min intervals were analyzed using repeated measures two-way analysis of variance. Post-hoc analyses were conducted using the Bonferroni test. Comparisons of data for the dose response representing total number of scratching bouts in 1 h were made using one-way analysis of variance followed by the Dunnett test. Data from two treatment groups were compared using the two-tailed t-test. The criterion for significance for all tests was set at p<0.05.

## Results

**Figure 1:**
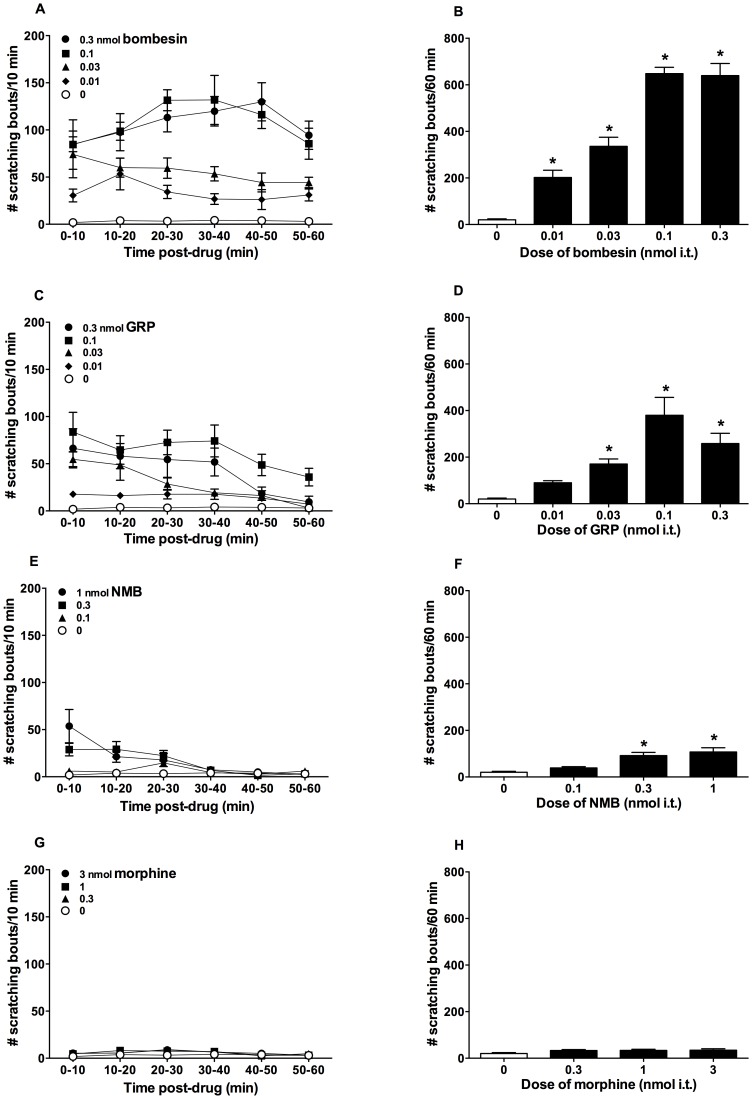
Effects of intrathecal administration of bombesin-related peptides and morphine on scratching behavior. Left panels show duration of scratching response and right panels show total number of scratching bouts for bombesin (A,B), GRP (C,D), NMB (E,F) and morphine (G,H). Mice were observed immediately after the intrathecal injections up to 1 h. Each value represents mean ± SEM (n = 6). Symbols represent different dosing conditions. An asterisk (*) represents significant difference from the vehicle controls (open bars; 0 µg) (P<0.05).

Figure 1 illustrates the duration and magnitude of scratching induced by intrathecal bombesin (0.01–0.3 nmol), GRP (0.01–0.3 nmol), NMB (0.1–1 nmol) and morphine (0.3–3 nmol) in mice observed for 1 h. Bombesin-related peptides, but not morphine, evoked scratching within 2 min after their administration. Mice treated with bombesin, GRP and NMB displayed other behaviors such as incessant facial grooming with forepaws and oral preening of the tail in addition to the scratching of the flank area by hindpaws as previously described [7], [24]. Bombesin elicited scratching in a dose-dependent manner [F(4, 25) = 63.2, p<0.05], and the scratching was maintained during the entire observation period of 1 h. GRP elicited scratching in dose-dependent [F(4, 25) = 11.8, p<0.05] and time-dependent [F(5, 150) = 7.3, p<0.05] manners lasting for 40 min. NMB evoked scratching in dose-dependent [F(3, 20) = 12.2, p<0.05] and time-dependent [F(5, 120) = 9.2, p<0.05] manners for 20 min. Minimum dose required to produce maximum scratching for bombesin and GRP was 0.1 nmol whereas for NMB, it was 1 nmol. At all doses tested, morphine-induced scratching was not significantly different from the vehicle condition [F(3,20 ) = 2, p>0.05].

**Figure 2:**
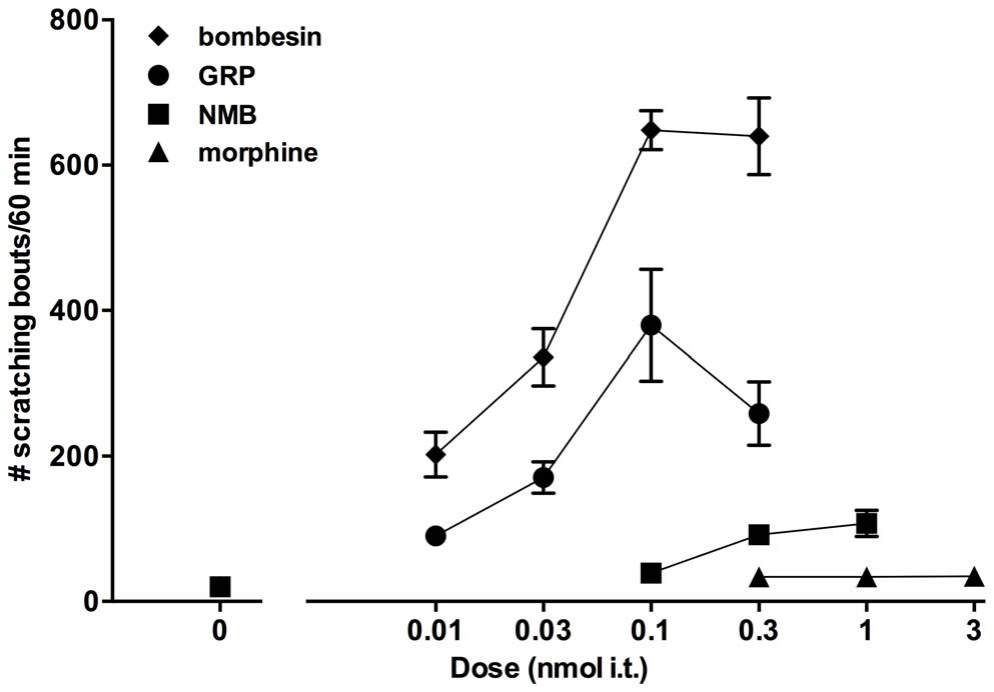
Comparison of dose response curves of intrathecal bombesin, GRP, NMB and morphine-induced scratching in mice. Each value represents mean ± SEM (n = 6) for number of scratching bouts observed for 1 h.

Figure 2 compares the dose response curves of scratching induced by intrathecally administered bombesin-related peptides and morphine. Bombesin and GRP showed similar potency to evoke scratching. However, the magnitude of scratching induced by bombesin was higher than that of GRP. NMB induced mild scratching and was less potent than bombesin and GRP. Morphine-induced scratching could not be distinguished from the vehicle.

**Figure 3:**
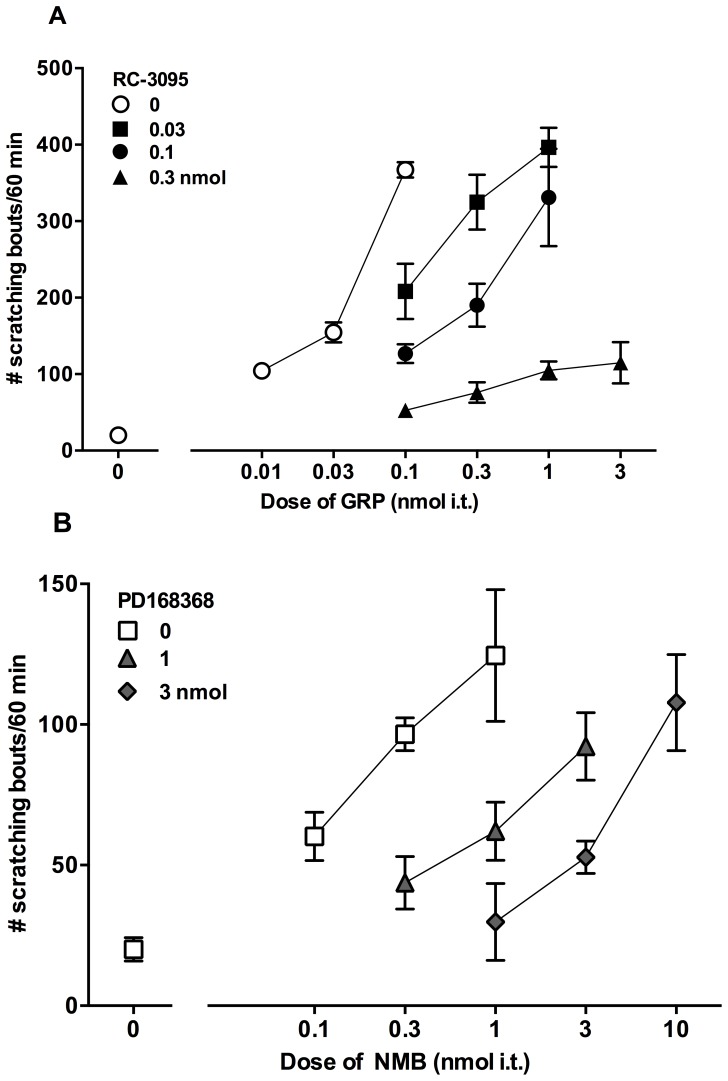
Effects of GRPr antagonist RC-3095 and NMBr antagonist PD168368 on intrathecal GRP- and NMB-induced scratching, respectively. Antagonists were administered intrathecally 10 min prior to GRP or NMB. Mice were observed immediately after the administration of GRP or NMB up to 1 h. Top panel shows changes in the dose response curve of GRP-induced scratching following RC-3095 pretreatment (A). Bottom panel shows changes in the dose response curve of NMB-induced scratching following PD168368 pretreatment (B). Each value represents mean ± SEM (n = 6) for number of scratching bouts observed across 1 h. Different symbols represent different dosing conditions.

Figure 3 illustrates the effects of intrathecally administered GRPr antagonist RC-3095 (0.03–0.3 nmol) and NMBr antagonist PD168368 (1–3 nmol) as a 10 min pretreatment on GRP and NMB-induced scratching, respectively. RC-3095 at 0.03 and 0.1 nmol, dose-dependently antagonized GRP-induced scratching as indicated by a 3 to 10 fold parallel rightward shift in the dose response curve of GRP. At 0.3 nmol of RC-3095, general suppression of scratching behavior was observed at all doses of GRP (0.1–3 nmol). PD168368 dose-dependently antagonized NMB-induced scratching as indicated by a 3 to 10-fold parallel rightward shift in the dose response curve of NMB. Vehicle pretreatment did not change the dose response curves for GRP or NMB.

**Figure 4:**
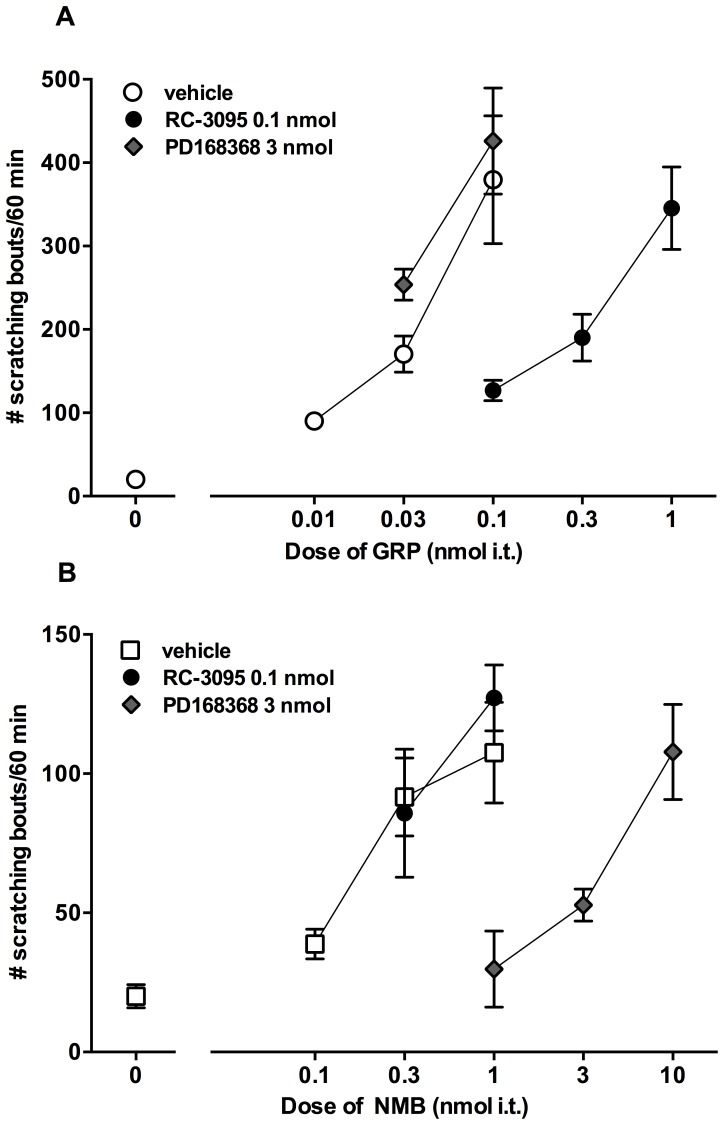
Cross examination of the effects of GRPr antagonist RC-3095 and NMBr antagonist PD168368 on intrathecal GRP- and NMB-induced scratching. Antagonists were administered intrathecally 10 min prior to GRP or NMB. Mice were observed immediately after the administration of GRP or NMB up to 1 h. Top panel shows changes in the dose response curve of GRP-induced scratching following pretreatment with active doses of PD168368 and RC-3095 (A). Bottom panel shows changes in the dose response curve of NMB-induced scratching following pretreatment with active doses of RC-3095 and PD168368 (B). Each value represents mean ± SEM (n = 6) for number of scratching bouts observed across 1 h. Different symbols represent different dosing conditions.

Figure 4 illustrates the effects of intrathecally administered PD168368 (3 nmol) on GRP-induced scratching and RC-3095 (0.1 nmol) on NMB-induced scratching as a 10 min pretreatment. Unlike RC-3095, PD168368 failed to cause a rightward shift in the dose response curve of GRP-induced scratching, thus maintaining the minimum dose of GRP (0.1 nmol) required to produce maximum scratching response. On the other hand, RC-3095 failed to cause a rightward shift in the dose response curve of NMB-induced scratching and maintained the minimum dose of NMB (1 nmol) required to produce maximum scratching response.

**Figure 5:**
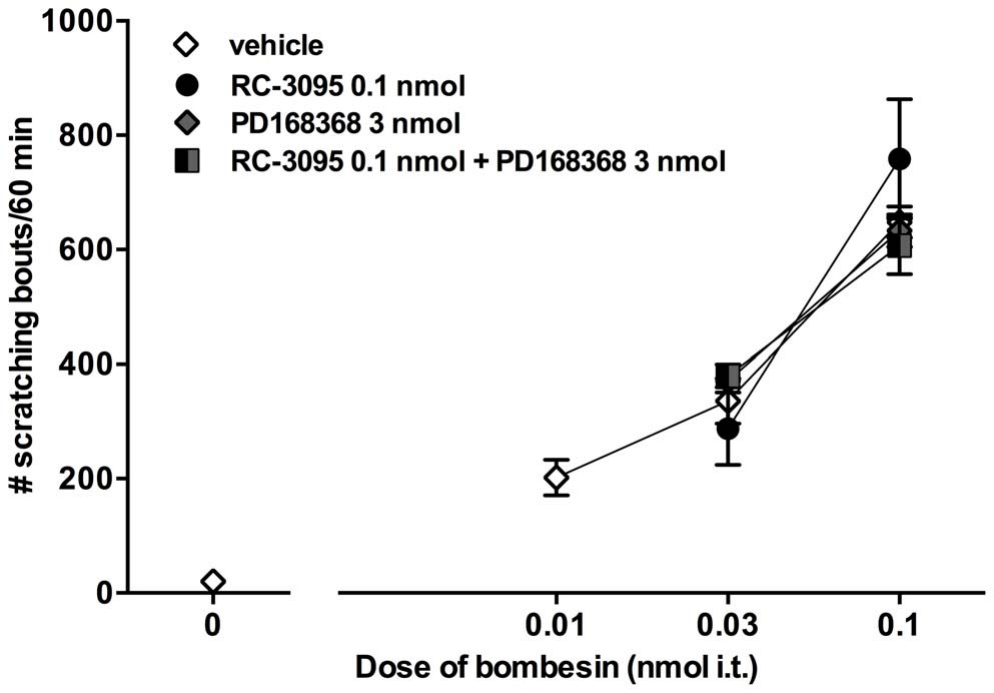
Effects of individual or co-administration of GRPr antagonist RC-3095 and NMBr antagonist PD168368 on the dose response curve of bombesin-induced scratching. Antagonists were administered intrathecally 10 min prior to bombesin. Mice were observed immediately after the administration of bombesin up to 1 h. Each value represents Mean ± SEM (n = 6) for number of scratching bouts. Different symbols represent different dosing conditions.

Figure 5 illustrates the effects of intrathecal administration of RC-3095 (0.1 nmol) or PD168368 (3 nmol) alone or their co-administration as a 10 min pretreatment on bombesin-induced scratching. As with the vehicle pretreatment, no change in the dose response curve of bombesin-induced scratching was observed following pretreatment with RC-3095, PD168368 or their combination. Magnitude and minimum dose of bombesin (0.1 nmol) required to produce maximum response did not change between antagonist and vehicle pretreatment groups.

**Figure 6:**
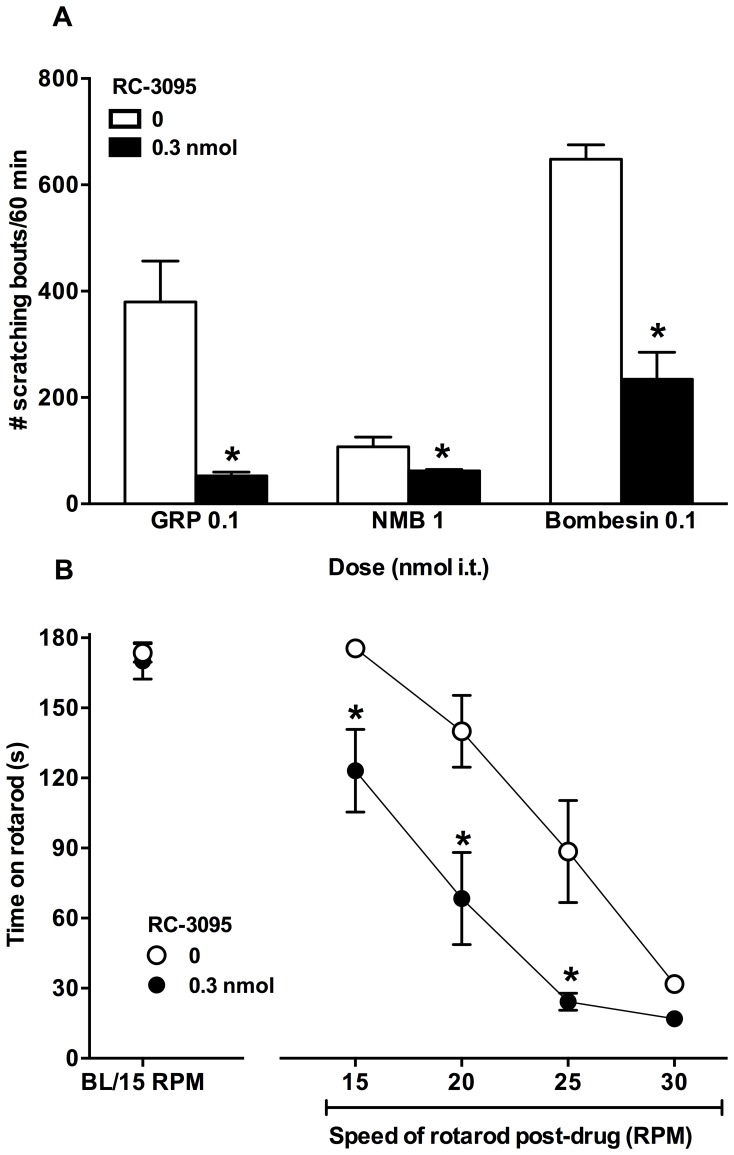
Effects of high dose of intrathecal RC-3095 on scratching induced by bombesin-related peptides and motor function. Top panel shows effects of RC-3095 on GRP, NMB and bombesin-induced scratching (n = 6) (A). Bottom panel shows effects of RC-3095 on the time spent by a mouse balancing on the rotarod (B). Mice (n = 10) were placed on the rotarod 10 min after the injection of RC-3095 and allowed to balance for 180 sec at different speeds. Different symbols represent different dosing conditions. Each value represents Mean ± SEM. An asterisk (*) represents significant difference from the vehicle controls (open bars or open circles; 0 µg) (P<0.05).

Figure 6 illustrates the effect of 0.3 nmol of RC-3095 on scratching-induced by bombesin-related peptides and motor function. RC-3095 significantly attenuated scratching induced by 0.1 nmol GRP [t(10) = 4.2, p<0.05], 1 nmol NMB [t(10) = 2.4, p<0.05] and 0.1 nmol bombesin [t(10) = 7.2, p<0.05]. Before the drug administration, all mice were able to balance on the rotarod at 15 RPM for approximately 180 sec. Mice treated with 0.3 nmol RC-3095 spent significantly less time on the rotarod at 15, 20, 25 and 30 RPM as compared to those which received the intrathecal injection of a vehicle [F(1,90) = 27.8, p<0.05].

## Discussion

Itch and pain are two independent somatosensory perceptions that elicit distinct behavioral responses but share many similarities in their neurotransmission. Itch signaling is thought to be driven by the activation of primary afferent nerve fibers or pruriceptors which send an input to a subpopulation of neurons in the superficial and deep dorsal horn in the spinal cord [25], [26]. In some cases such as those of neurogenic or psychogenic origin, itch can also be originated in the spinal cord [2]. Interestingly, the subpopulation of neurons in the spinal cord dorsal horn that is excited by pruritogens, also responds to noxious nociceptive stimuli in rodents and primates [27]–[29]. Recently it was shown that selective ablation of bombesin-recognized neurons in lamina 1 of dorsal spinal cord markedly attenuated scratching evoked by several pruritogens but did not affect nociceptive responses in mice [30]. This raises a possibility that the spinal receptors for bombesin-related peptides may exclusively regulate itch neurotransmission and need further investigation for the identification of novel pharmacological targets to block pruritus.

The first part of the study determined the basic characteristics of scratching induced by intrathecally administered bombesin, GRP and NMB in mice. By testing multiple doses, this study established dose response curves for bombesin, GRP and NMB and identified minimum dose of each peptide required to produce maximum scratching response. All three peptides elicited scratching dose dependently with different degree and duration of scratching activity. Bombesin evoked most profound scratching response that lasted over 1 h, followed by GRP which evoked robust response for 40 min whereas NMB induced mild scratching which lasted for 20 min. It is possible that the three peptides have different rates of proteolytic degradation, which might lead to the different durations of action. Such differences in the duration and magnitude of bombesin, GRP and NMB following spinal and supraspinal administration have been previously documented in rodents [13], [14], [18].

Itch is one of the most prevalent and severe side effects of spinally administered MOP agonists like morphine and DAMGO, which also elicit long lasting profound scratching in monkeys at the antinociceptive doses, as seen in human subjects [31]–[33]. Antagonist studies reveal that in primates, intrathecal morphine-induced itch is mediated by selective activation of MOP but not other opioid receptor subtypes [32]. In addition to attenuating MOP-mediated itch, MOP antagonists have also been used to treat itch caused by liver diseases like cholestasis [34], [35]. This indicates that itch neurotransmission is at least in part driven by the endogenous opioids. However, other neurotransmitters of itch may be involved. Therefore, it is important to investigate whether other itch mediators like bombesin-related peptides and their receptors elicit profound scratching like morphine in animals. In the present study, effects of intrathecal morphine at antinociceptive doses on scratching behavior were determined in mice [36], [37]. However, morphine failed to elicit scratching in mice that could be distinguished from the intrathecal vehicle injection. Inability of intrathecal morphine to induce profound scratching has been previously documented in rats [9], although a few studies have reported some scratching activity in response to intrathecal morphine in mice [17], [22]. However, both the magnitude and duration of this scratching activity (i.e., total ∼20–30 bouts lasting 10–15 min) are very small as compared to the non-opioid peptides like GRP (∼400 bouts lasting 40 min) or bombesin (∼700 bouts lasting over 60 min) suggesting the dramatic differences in the scratching activity elicited by different compounds in the same species. On the other hand in monkeys, antinociceptive doses of intrathecal morphine elicited intense scratching response (>3500 scratches lasting over 6 h) [33] indicating that species differences affect the ability of intrathecal morphine to evoke scratching. It is not entirely clear why the rodents, unlike humans and monkeys, are insensitive to intrathecal opioid-induced scratching. It is possible that in rodents, the neurocircuitry modulating intrathecal opioid-induced antinociception may be independent of the itch neurotransmission, i.e. spinal MOP receptors may play a role in driving antinociception but cannot concomitantly elicit the scratching behavior in rodents. It has been demonstrated that there is a subset of inhibitory interneurons regulating itch in the dorsal horn of mouse spinal cord [38]. It is important to compare these inhibitory circuits between rodents and primates in the dorsal horn that may mediate cross-inhibition between itch and pain modalities. On the other hand, supraspinal administration of bombesin elicits intense scratching in both rodents and monkeys [7], [9], [18]. However, ability of intrathecally administered bombesin-related peptides to evoke scratching response remains to be documented in monkeys. Therefore, attributed to the species differences, rodent models may not be ideal to study intrathecal opioid-induced itch but can be well utilized to investigate the mechanisms underlying non-opioid (e.g. GRPr) mediated itch scratching.

Second part of the study determined the independent role of spinal GRPr and NMBr in GRP and NMB-induced scratching using intrathecal administration of selective GRPr antagonist RC-3095 and selective NMBr antagonist PD168368. Pretreatment with RC-3095 (0.03–0.1 nmol) dose dependently caused a 3 to 10-fold parallel rightward shift in the dose response curve of GRP-induced scratching indicating that the antagonism was competitive and reversible at GRPr. Thus, GRP-induced scratching was due to the selective activation of GRPr. Similarly, NMB-induced scratching was mediated by the selective activation of NMBr. Interestingly, these active doses of RC-3095 and PD168368 when cross-examined against NMB and GRP, no change in the dose response curves of NMB or GRP was observed. This indicates that GRPr do not mediate NMB-induced scratching and *vice versa*. Previous studies using intracerebroventricular administration have documented such independent mechanisms of both supraspinal GRP and NMB to elicit scratching in rats [18]. These studies demonstrate that both GRPr and NMBr in the central nervous system of rodents independently regulate itch scratching behavior regardless of spinal and supraspinal regions.

Bombesin has high affinity for GRPr and NMBr (4–34 nM) [15]. To determine if GRPr and NMBr mediate bombesin-elicited scratching, active doses of RC-3095 and PD168368 were tested alone or in combination against bombesin. However, no change in the dose response curve of bombesin-elicited scratching was observed, indicating that bombesin does not elicit scratching via GRPr or NMBr. Similarly, at the supraspinal level, active doses of RC-3095 and PD168368 failed to reduce bombesin-induced scratching in rats [18]. MOP, delta and kappa-opioid receptor antagonists have also failed to attenuate scratching induced by centrally administered bombesin [9], [39]. Therefore, it is possible that bombesin acts via independent, yet unidentified subset of receptors to induce scratching. No effective bombesin-blocking agent is currently available. Although, [desTrp^3^,Leu^8^]phyllolitorin, a phyllolitorin analog, was able to block supraspinal bombesin-induced scratching, it does not have measurable binding affinity at bombesin receptors [40]. Nevertheless, scratching induced by central administration of bombesin is a valuable experimental approach to assess potential antipruritic drugs. Together, these findings indicate that there are unidentified receptor mechanisms that drive bombesin-induced scratching in rodents.

Recent studies raised the possibility that GRP receptors in the spinal cord are the key mediators of itch sensation. Genetic and pharmacological blockade of GRPr in mice attenuated, but did not completely block, scratching induced by intradermally administered non-histaminergic pruritogens [16]. Ablation of bombesin-recognized neurons in the spinal cord, which also include GRPr expressing neurons, attenuated scratching induced by intradermally administered pruritogens irrespective of their histamine dependency [30]. This suggests that there may be additional mechanisms other than GRPr that drive the itch scratching. Although blockade of GRPr caused reduction in the mild to moderate scratching induced by intradermal pruritogens, GRPr antagonists failed to attenuate *profound* scratching induced by other ligands like the kappa opioid receptor antagonist 5′-guanidinonaltrindole [24] and bombesin [18]. In the present study, attenuation of bombesin and NMB-induced scratching was observed with the high dose of RC-3095 (0.3 nmol). However, at this dose RC-3095 caused a general suppression of GRP-induced scratching in absence of the parallel rightward shift. It should be noted that this type of antagonism signifies a noncompetitive binding of RC-3095 that is not selective to GRPr and/or could have unspecified behavioral toxicity. When the mice treated with high dose of RC-3095 were tested on the rotarod for their motor function, their ability to remain on the rotarod was compromised. In other words, GRPr antagonist only attenuated scratching at doses that also interfered with the motor function. Expression of GRP in the motor areas of lumbosacral spinal cord and reduced locomotor activity in GRPr deficient mice has been previously reported [41], [42]. Therefore, GRPr is only one of the key mediators of itch and may have a selective role in regulating some but not all types of itch. Nevertheless, it is worth evaluating GRPr and NMBr antagonists in animal models of chronic itch such as atopic dermatitis and cholestasis.

Overall, the present study compared characteristics of spinally administered bombesin-related peptides versus morphine for eliciting scratching in mice. Vast differences observed in the magnitude of scratching induced by morphine versus bombesin, GRP and NMB suggested that rodents may not be the ideal species to examine pruritus induced by intrathecal opioids. This study is the first to provide detailed pharmacological evidence that spinal GRPr and NMBr independently drive scratching whereas bombesin elicits scratching through receptor mechanisms independent of GRPr and NMBr. Most importantly, GRPr antagonists at functionally receptor-selective doses can block only the spinal GRP-elicited scratching. At higher doses, GRPr antagonists may generally suppress scratching mediated by different receptors, but it could be confounded by the nonselective behavioral effects in mice such as impairment of motor function. Together, the present study not only improves the understanding of itch neurotransmission in the spinal cord but also lays out the pharmacological basis for the development of GRPr and NMBr antagonists for the treatment of pruritus.
